# Microfluidic screening and genomic mutation identification for enhancing cellulase production in *Pichia pastoris*

**DOI:** 10.1186/s13068-022-02150-w

**Published:** 2022-05-14

**Authors:** Huiling Yuan, Ying Zhou, Yuping Lin, Ran Tu, Yufeng Guo, Yuanyuan Zhang, Qinhong Wang

**Affiliations:** 1 CAS Key Laboratory of Systems Microbial Biotechnology, Tianjin Institute of Industrial Biotechnology, Chinese Academy of Sciences, Tianjin, 300308 China; 2 National Center of Technology Innovation for Synthetic Biology, Tianjin, 300308 China; 3 University of Chinese Academy of Sciences, Beijing, 100049 China; 4 Wuhan Sunhy Biology Co., Ltd, Wuhan, 430206 China

**Keywords:** Cellulase, *Pichia pastoris*, High-throughput screening (HTS), Whole-cell directed evolution, Droplet-based microfluidic, Whole-genome resequencing

## Abstract

**Background:**

*Pichia pastoris* is a widely used host organism for heterologous production of industrial proteins, such as cellulases. Although great progress has been achieved in improving protein expression in *P. pastoris*, the potential of the *P. pastoris* expression system has not been fully explored due to unknown genomic impact factors. Recently, whole-cell directed evolution, employing iterative rounds of genome-wide diversity generation and high-throughput screening (HTS), has been considered to be a promising strategy in strain improvement at the genome level.

**Results:**

In this study, whole-cell directed evolution of *P. pastoris*, employing atmospheric and room temperature plasma (ARTP) mutagenesis and droplet-based microfluidic HTS, was developed to improve heterogenous cellulase production. The droplet-based microfluidic platform based on a cellulase-catalyzed reaction of releasing fluorescence was established to be suitable for methanol-grown *P. pastoris*. The validation experiment showed a positive sorting efficiency of 94.4% at a sorting rate of 300 droplets per second. After five rounds of iterative ARTP mutagenesis and microfluidic screening, the best mutant strain was obtained and exhibited the cellulase activity of 11,110 ± 523 U/mL, an approximately twofold increase compared to the starting strain. Whole-genome resequencing analysis further uncovered three accumulated genomic alterations in coding region. The effects of point mutations and mutant genes on cellulase production were verified using reconstruction of point mutations and gene deletions. Intriguingly, the point mutation Rsc1^G22V^ was observed in all the top-performing producers selected from each round, and gene deletion analysis confirmed that Rsc1, a component of the RSC chromatin remodeling complex, might play an important role in cellulase production.

**Conclusions:**

We established a droplet-based microfluidic HTS system, thereby facilitating whole-cell directed evolution of *P. pastoris* for enhancing cellulase production, and meanwhile identified genomic alterations by whole-genome resequencing and genetic validation. Our approaches and findings would provide guides to accelerate whole-cell directed evolution of host strains and enzymes of high industrial interest.

**Supplementary Information:**

The online version contains supplementary material available at 10.1186/s13068-022-02150-w.

## Background

Cellulase can catalyze the hydrolysis of cellulose into fermentable monosaccharides and has numerous applications in biofuel production, textile polishing and finishing, paper, laundry, food, and feed processing industries [1–3]. Currently, filamentous fungi genera, such as *Trichoderma*, *Penicillium*, and *Aspergillus*, are the major source of commercial cellulase mixtures [4–6]. However, the end-product inhibition to cellulase expression and activity still remains a challenge [7]. Therefore, to address this obstacle, heterologous expression of cellulase genes in non-cellulolytic microorganisms becomes a promising strategy [8–10]. Yeast, a simple and effective single-cell eukaryotic organism, is frequently harnessed as a recombinant protein expression system. Among yeast species, the methylotrophic yeast *Pichia pastoris* (syn. *Komagataella phaffii*) has become a widely used host organism for heterologous protein production, and the *P. pastoris* expression technology has been commercially available for many years [11–15]. The success of the *P. pastoris* expression system can be attributed to several advantages: (i) it can be grown to very high cell densities in inexpensive media by relatively easy manipulation of fermentation parameters; (ii) it has a high-level secretion system of heterologous proteins; (iii) it has strong and tightly regulated promoters; and (iv) it can perform higher eukaryotic protein post-translational modifications [16]. To date, more than 5000 proteins have been expressed in *P. pastoris* systems [15]. By applying various rational and irrational engineering strategies, such as promoters, signal peptides, and cultivation strategies, significant progress has been achieved in improving cellulase expression by *P. pastoris* [17, 18]. For instance, *P. pastoris* was used for expression of a *Sclerotinia sclerotiorum* GH45 endoglucanase, showing high stability over a wide pH range and thermostability [19]. The marketed PichiaPinkexpression system (Life Technologies Corporation, CA, USA) of *P. pastoris* was optimized for expression of two fungal endoglucanases and yielded up to 5 g/l total secreted protein [20]. A cellulase enzyme HT639plus production by *P. pastoris* has been developed and commercialized by Sunhy Corporation (Hubei, China) [21]. However, it is still hard to further improve recombinant protein production on the basis of high expression levels in industrial strains already in use, which is mostly due to unknown impact factors hidden in its genome [22]. Thus, it is worthy of generating genome-wide mutagenesis and more efficient screening methods, thus discovering more genomic variants associated with heterogenous protein expression in *P. pastoris*.

Whole-cell directed evolution, which employs iterative rounds of diversity generation and screening, is considered as a promising strategy to develop desirable production traits at the genome-wide level [23–25]. Genome-wide diversity generation approaches have been developed to be more efficient and well applied, such as irrational methods including UV mutagenesis, EMS chemical mutagenesis, genome shuffling, atmospheric and room temperature plasma (ARTP) mutagenesis, etc., and rational methods including site-directed RNAi, CRISPR-Cas, recombineering, and transposon insertion, etc. [26–32]. By contrast, the lack of suitable high-throughput screening (HTS) methods is still a primary bottleneck [33, 34]. Conventional agar plate- and microtiter plate-based assays are the most widely used screening formats. However, the screening throughput of these formats is limited, and the process is laborious [35]. Fluorescence-activated cell sorting (FACS) can screen individual cells at a rate of up to 10^8^ cells per day, but can not be used for secreted metabolites and enzymes. In recent years, droplet-based microfluidics has emerged as a powerful HTS method because of its significantly higher throughput and lower reagent consumption compared to MTP, and its compartmentalization assays in emulsion droplets compared to FACS [36–38]. In this approach, monodisperse picolitre water-in-oil droplets are generated and manipulated (such as droplet splitting, fusion, picoinjection, detection, and sorting) at rates of thousands per second. These droplets can be used as microreactors to compartmentalize individual samples (cell, DNA, protein, chemical/biological reaction, etc.) for reliable quantitative analysis. Through compartmentalization, droplets provide a high-performance platform to enhance the linkage of phenotype and genotype during whole-cell directed evolution [39]. In the past few years, droplet microfluidics has been successfully applied to directed evolution of different microorganisms, including bacteria, yeast, and filamentous fungi, which expressed different enzymes including β-galactosidase, cellulase, α-amylase, aldolase, and esterase [35, 40–45].

**Fig. 1:**
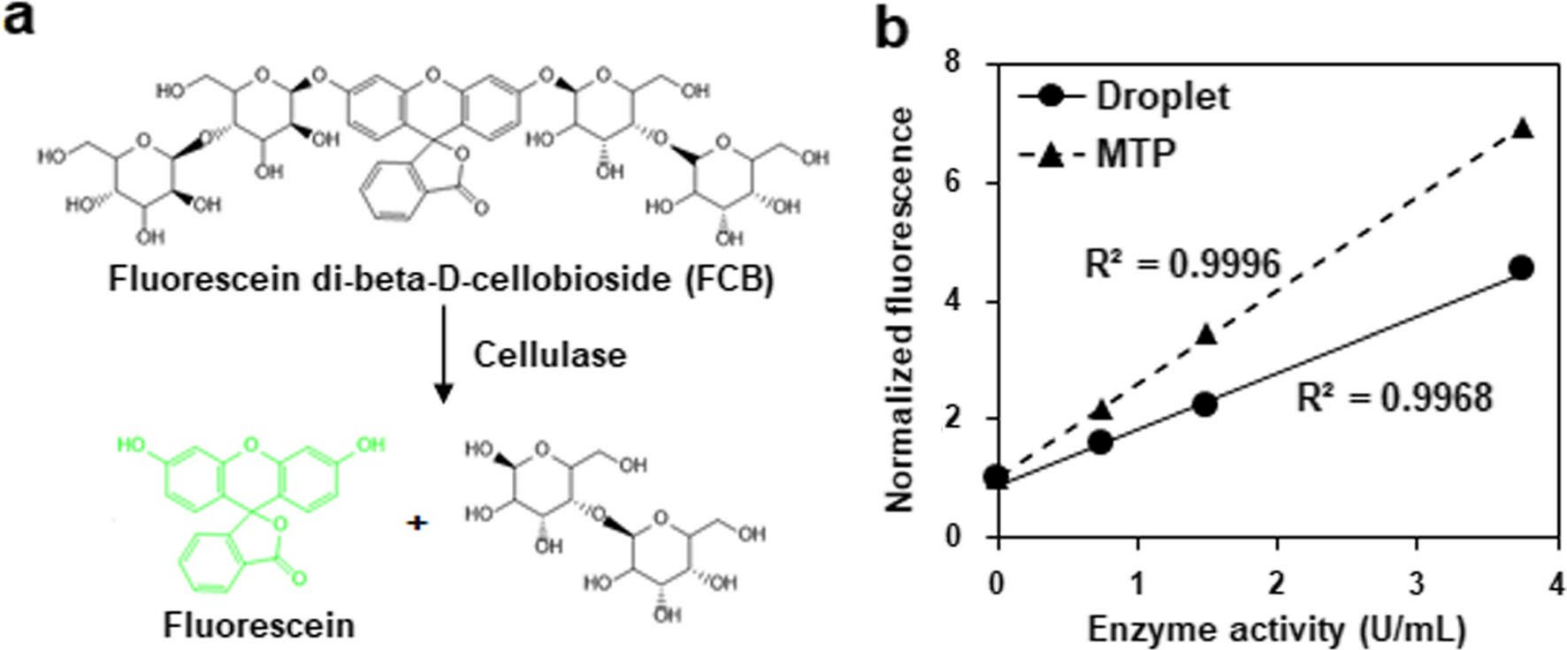
Cellulase activity measurement using fluorescein di-beta-D-cellobioside (FCB). **a** Mechanism of fluorescence-based cellulase activity measurement. Green fluorescent compound fluorescein can be released from FCB by cleavage of the β-1,4-D-glycosidic bonds under cellulase treatment. **b** Fluorescence-based cellulase activity assay using droplet (solid line) and MTP (dashed line) formats

In this study, to further enhance heterogenous protein production performances of an industrial recombinant cellulase-producing *P. pastoris* strain, we developed a droplet-based microfluidics HTS system according to a cellulase-catalyzed reaction of releasing fluorescence (Fig. 1a), combined with ARTP mutagenesis method, to conduct whole-cell directed evolution. The droplet-based microfluidic platform including droplet generation, off-chip incubation, reinjection, and sorting, was established to be suitable for methanol-grown *P. pastoris*. After five rounds of evolution, cellulase production of the selected mutant strains were gradually improved compared to the starting strain. Furthermore, some key genomic impact factors affecting heterogenous protein expression were demonstrated by genome resequencing analysis and genetic validation. Taken together, this study developed and tested a high-throughput and efficient strategy of enhancing heterogenous cellulase production in *P. pastoris*, thus providing more guidance to accelerate whole-cell directed evolution of host strains and enzymes of high industrial interest.

## Results

### Establishment of microfluidic screening for cellulase-producing *P. pastoris*

To monitor cellulase activity in microfluidic droplets, a fluorescence-based method using a fluorogenic substrate fluorescein di-beta-D-cellobioside (FCB) was employed [46]. Cellulase can convert FCB into the compound fluorescein with green fluorescence (Fig. 1a). To evaluate the performance of this FCB-based cellulase activity measurement method, different concentrations of commercial cellulase HT639plus (Wuhan Sunhy Biology Co., Ltd, Wuhan, China) were incubated with FCB for 48 h in droplets using microtiter plate (MTP) as a control [21], and subjected to fluorescence detection. As shown in Fig. 1b, for the samples with same enzyme activities, higher fluorescence intensities were detected by the MTP assay format than those by the droplet assay format. This difference might be due to different detection volumes and instruments were used. Nevertheless, both assay formats indicated positive correlations between cellulase activities at the range of 0–4 U/mL and fluorescence intensities.

**Fig. 2:**
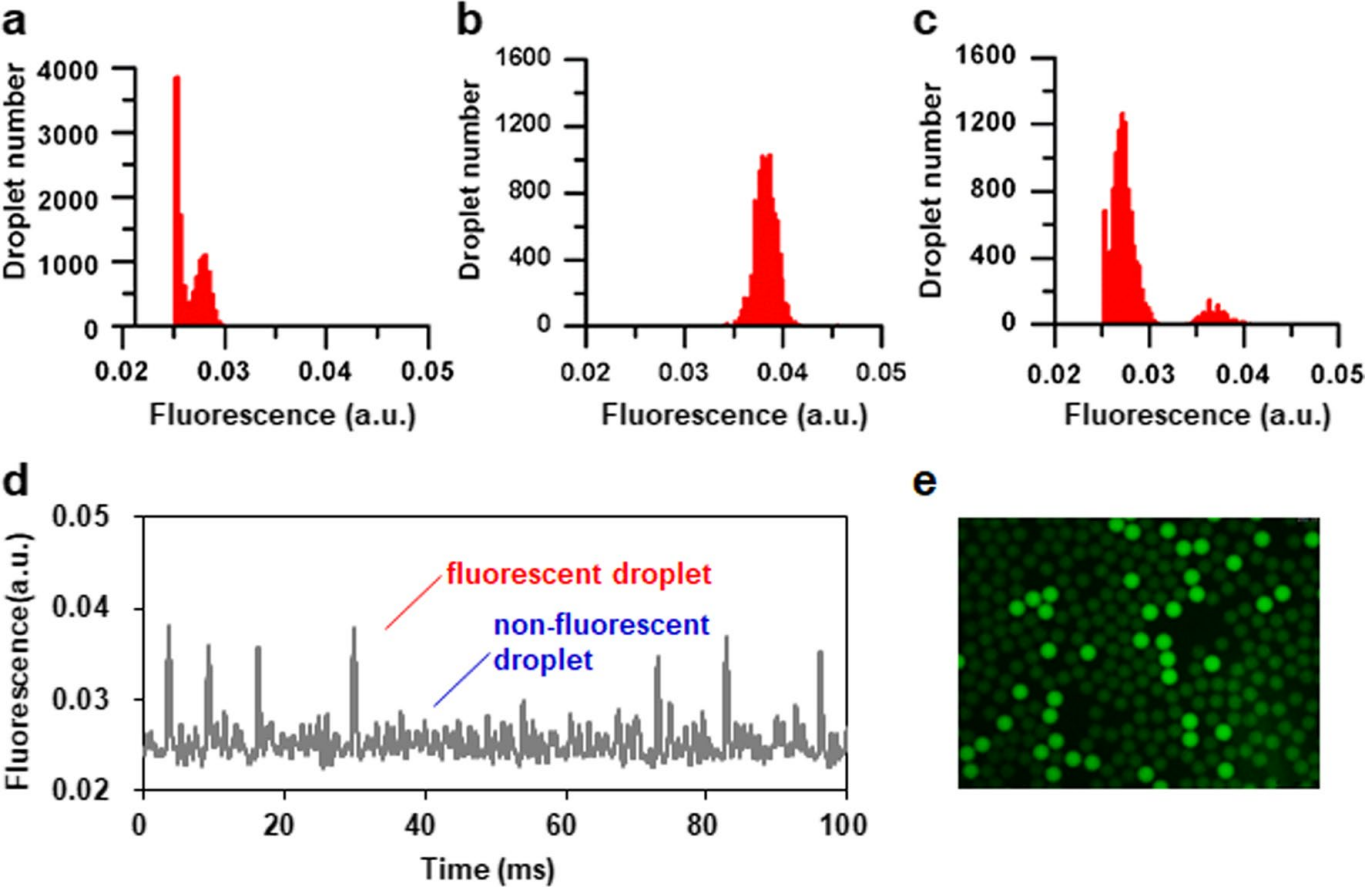
Effect of methanol on fluorescein leakage in droplets. **a** Histogram of nonfluorescent droplets only containing BMMY medium with 1% methanol. **b** Histogram of fluorescent droplets generated from 40 h reaction solution of cellulase in BMMY medium with 1% methanol and 20 μM fluorogenic substrate FCB. **c** Histogram of a binary mixture of nonfluorescent and fluorescent droplets at a 10:1 mixing ratio. **d** A typical recording of fluorescence signals when detecting the binary droplet mixture. **e** Fluorescence microscopy image of the binary droplet mixture. Droplets were incubated at 30 °C for 48 h. All histograms show approximately up to 10,000 droplets

Methanol is usually used as the sole carbon source and inducer for protein expression by recombinant *P. pastoris* strains. Furthermore, the retention of fluorescent marker in water-in-oil droplets is important to the precision of fluorescence-activated droplet sorting. However, the effects of methanol, as an organic solvent, on the stability and fluorescence leakage of the water-in-oil droplet have not yet been evaluated. First, therefore, the droplets containing different concentrations of methanol (0 to 2%, v/v) were formed, and the diameters of droplets were measured by image analysis over time. The results showed that the sizes of droplets have no obvious changes during 48 h incubation (Additional file 1: Fig. S1a). Furthermore, microscopy images showed that the droplets still maintained a high degree of monodispersity (Additional file 1: Fig. S1b). Second, to test the effect of methanol on fluorescence leakage, three types of droplets, including nonfluorescent droplets only with BMMY medium, fluorescent droplets with cellulase and FCB, and the binary mixture of nonfluorescent and fluorescent droplets with a 10:1 mixing ratio, were generated, incubated for 48 h, and thereafter subjected to microfluidic measurement of their fluorescence distributions. Compared to the fluorescence distributions of nonfluorescent droplets and fluorescent droplets, histogram of the binary droplet mixture showed a clear separation of nonfluorescent and fluorescent droplets (Fig. 2a–c). During the microfluidic detection, the fluorescence signals of nonfluorescent and fluorescent droplets can be well distinguished (Fig. 2d), which was further confirmed by microscope image of droplets (Fig. 2e). Taken together, methanol, which is required for protein expression by recombinant *P. pastoris* strains, would have no obvious effect on subsequent microfluidic screening.

**Fig. 3:**
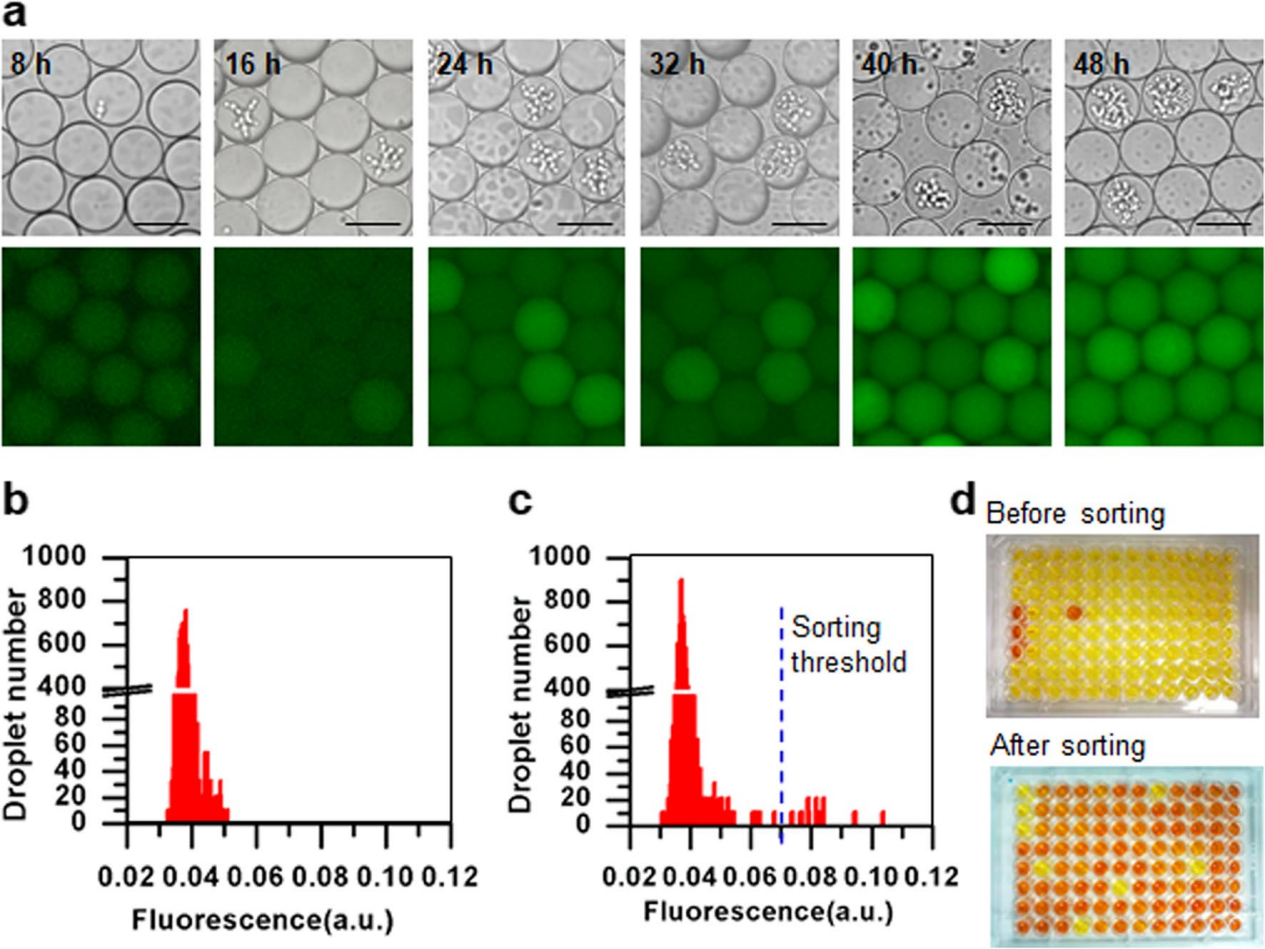
Establishment of the fluorescence-based droplet microfluidic screening for cellulase-producing *P. pastoris* cells. **a** Fluorescence signal detection of cellulase-producing cells of the *P. pastoris* strain SHY169 (green fluorescence-positive strain) in droplets at different timepoints. Scale bar: 50 μm. The exposure time was 50 ms. **b** Fluorescence histogram of droplets at 0 h. The mixture of cellulase-producing and non-cellulase producing cells at a 1:99 ratio was used for droplet generation. **c** Fluorescence histogram of droplets after incubation for 40 h at 30 °C. The blue dashed line indicates the sorting threshold. **d** Cellulase activity measurement of the selected cells using the DNS method. The first three wells in the first column were negative controls of non-cellulase producing cells from the strain GS115, and the next three wells were positive controls of cellulase-producing cells from the strain SHY169

To determine the optimal incubation time for droplet detection and sorting, the cellulase-producing *P. pastoris* strain SHY169 and FCB were encapsulated in droplets, and their green fluorescence signals were detected by fluorescence microscopy at different timepoints. After 24 h of cultivation, the signals became detectable, and the signal intensities continued to increase until 40 h (Fig. 3a). Thus, we selected 40 h as the incubation time for droplet sorting.

Finally, we validated whether the established droplet-based microfluidic platform can be used to sort out *P. pastoris* cells with cellulase production. A sorting experiment was conducted to enrich cellulase-producing SHY169 cells from the artificially mixed library, which was composed of SHY169 and non-cellulase producing GS115 at a 1:99 ratio. The histogram of background fluorescence from droplets at the 0-h timepoint showed only one peak centered on the fluorescence (a.u.) of 0.04 (Fig. 3b). After 40-h incubation, droplets with higher fluorescence were differentiated from the background fluorescence (Fig. 3c), and the 0.1% most fluorescent droplets were sorted out by setting a threshold at the fluorescence (a.u.) of 0.07. Ninety cells from the selected droplets were further validated for cellulase production capacities using flask experiments, and cellulase activity was measured by the 3,5-dinitrosalicylic acid (DNS) reduction method. Meanwhile, ninety cells from the original mixed library were used as a control. Before sorting, one in ninety cells showed cellulase activity, while 85 in 90 cells showed cellulase activity after sorting (reds well in Fig. 3d except for negative and positive controls in the first six wells in the first column), resulting in about 94.4% positive sorting efficiency and representing enrichment ratio of 1513 [36]. These results indicated that our droplet-based microfluidic platform could be efficient for the high-throughput sorting of cellulase-producing cells.

**Fig. 4:**
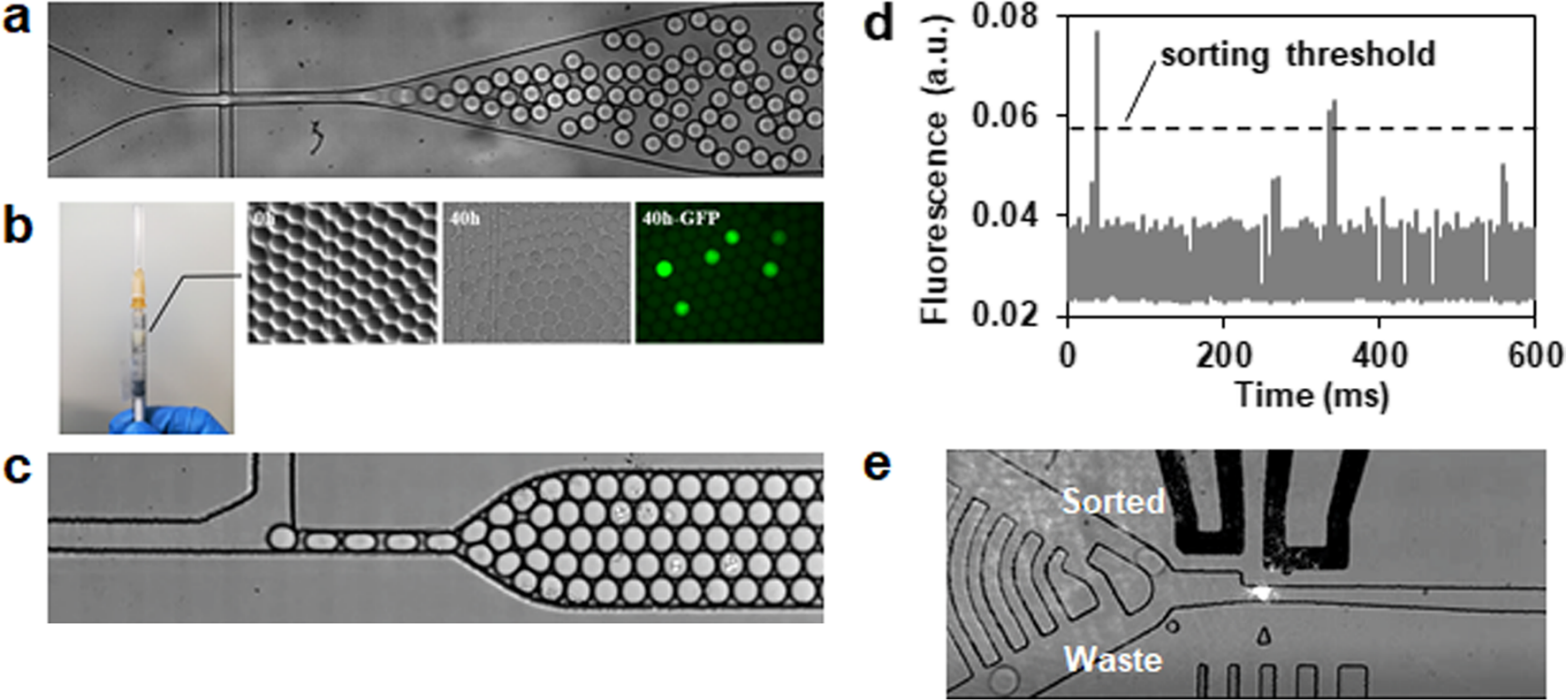
Workflow of the droplet-based microfluidic screening. **a** Droplet generation. Cells were encapsulated in microdroplets together with the substrate FCB using an appropriate cell to droplet ratio. **b** Droplet incubation. The emulsion was incubated off-chip in a syringe to allow cell growth, cellulase expression, and fluorescence signal development. **c** Droplet reinjection. After incubation, the emulsion droplets were reinjected into a sorting device. **d** Droplet fluorescence detection. The fluorescence signal of each droplet was detected and analyzed by the optical setup. **e** Droplet sorting. Droplets were sorted based on resulting fluorescence using dielectrophoresis

Overall, the droplet-based microfluidic platform was established as follows (Fig. 4): (a) encapsulation of single cells together with a fluorogenic substrate FCB in 10 pL droplets (Fig. 4a); (b) off-chip incubation for cell growth and cellulase expression for 40 h at 30 °C (Fig. 4b); (c) reinjection of droplets for analysis and sorting based on the fluorescence signal, where about 2 × 10^6^ droplets were sorted at a throughput of 300 droplets per second and 0.01% to 0.03% droplets with positive signals were collected (Fig. 4c–e); and (d) recovery of cells from the sorted droplets.

### Microfluidic screening of enhanced cellulase production from ARTP mutagenesis libraries

**Fig. 5:**
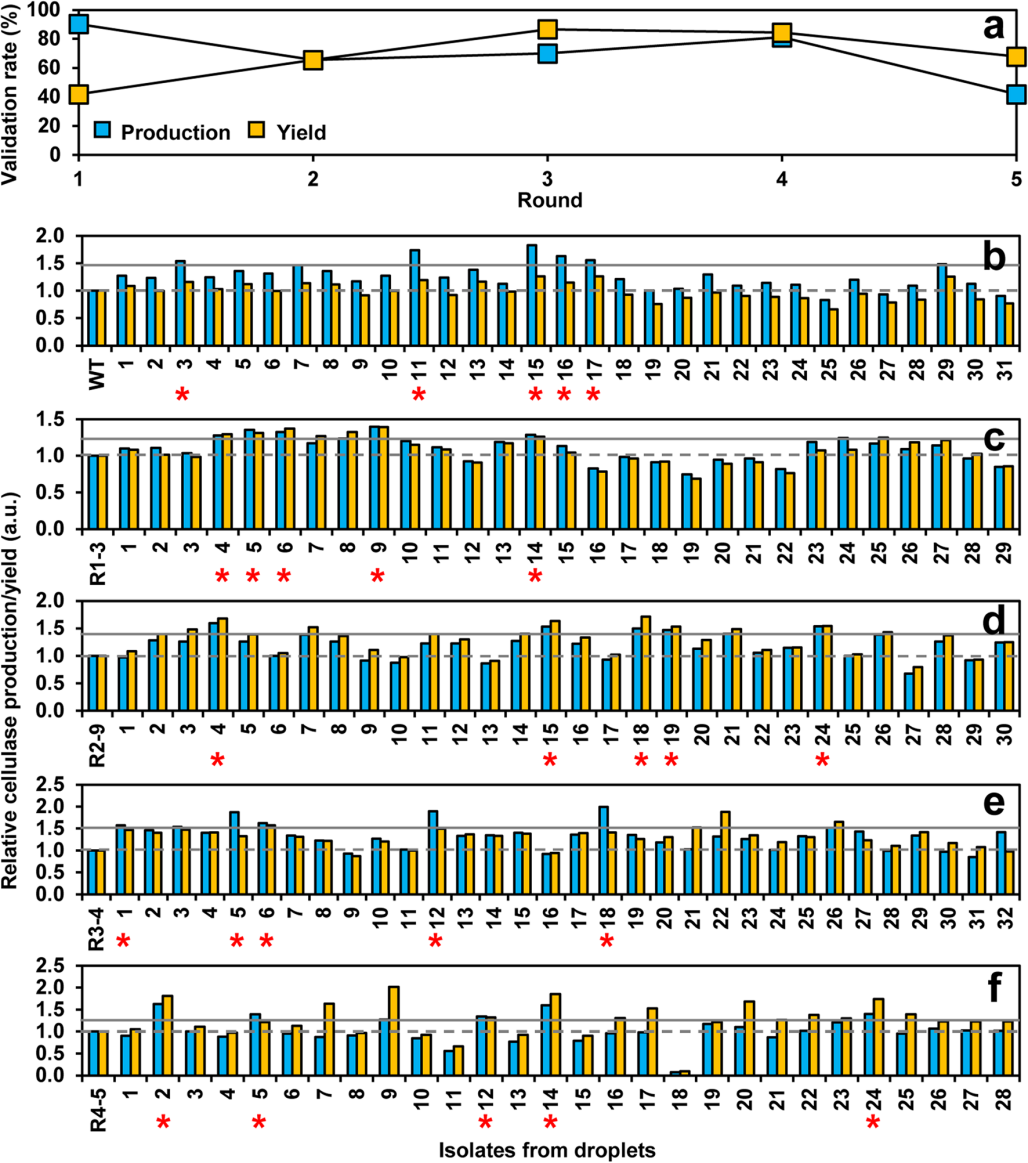
Primary validation of cellulase production performance by isolates from five iterative ARTP mutagenesis and screening. **a** Validation rates. They were calculated as the percentage of isolates with higher cellulase production and yield compared to the starting strain among the tested isolates sorted from droplets. Relative cellulase production and yield data were from Fig. 5b to 5f. **b** First round of mutagenesis and screening. The wild-type cellulase-producing strain SHY169 was used as a starting strain. **c** Second round. **d** Third round. **e** Fourth round. **f** Fifth round. In **c** to **f**, the best cellulase producer was selected from the last round to be a starting strain for the next round. About thirty clones were randomly picked from the recovery plates, and their cellulase production was evaluated by flask fermentation. Production is measured in units of cellulase activity per volume and the yield is cellulase activity per 1 OD_600_ unit of cells. Data were normalized to the starting strain of each round. The solid and dashed lines indicate the thresholds of strain selection for production and yield, respectively. Top five cellulase hyperproducers from each round were indicated by red stars

We next applied the droplet-based microfluidic system for screening cellulase hyperproducers from ARTP-mutagenized *P. pastoris*. To increase the chance of obtaining desirable mutants, five rounds of iterative ARTP mutagenesis and screening were conducted. For each round, the best cellulase producer was selected to be a starting strain for the next round. Microfluidic screening was performed by following the above workflow (Fig. 4). From each round, 200 to 600 droplets with positive green fluorescence were sorted out and subjected to the subsequent primary, secondary and final validations by flask fermentation and 5-L bioreactor fermentation for the best producer. Cellulase production performance of cells was evaluated by two parameters including production and yield. Production is measured in units of cellulase activity per volume, whereas yield is measured in units of cellulase per OD_600_ of biomass. First, about thirty clones from each round were randomly picked from the recovery plates for primary validation (Fig. 5). Validation rate was calculated as the percentage of isolates with higher cellulase production and yield compared to the starting strain. In terms of cellulase production, the first round showed the highest validation rate of 90%, while the fifth round showed the lowest validation rate of 42% (Fig. 5a). In terms of cellulase yield, the first round showed the lowest validation rate of 42%, while the third round showed the highest validation rate of 87% (Fig. 5a). Overall, an average validation rate was around 70%. Furthermore, compared to the starting strain, cellulase production in each of the five rounds were maximumly increased 1.8, 1.4, 1.6, 2.0, and 1.6-fold, respectively, whereas cellulase yield were maximumly increased 1.3, 1.4, 1.7, 1.9, and 2.0-fold (Fig. 5b–f). These results indicated that the iterative ARTP mutagenesis and screening could be an efficient way to improve and enrich cellulase production performance. In addition, there was no apparent positive relationship between cellulase production and yield for the same isolate. Thus, we selected five isolates from each round, which showed top performance at cellulase production and no decrease in cellulase yield, to be further evaluated (Fig. 5b–f).

**Fig. 6:**
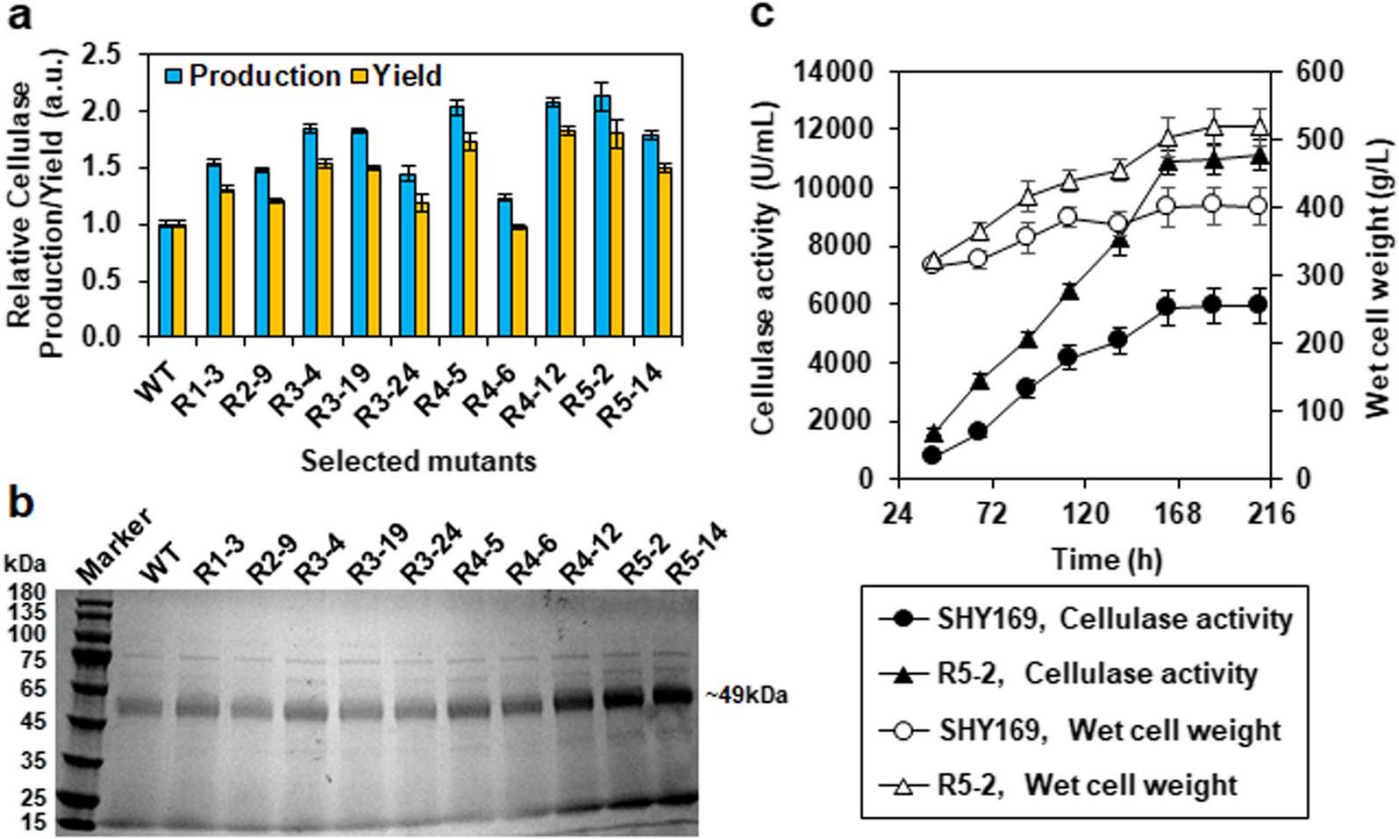
Enhanced cellulase production by selected mutants. **a** Final validation of the selected mutants for cellulase production by flask fermentation. Data were normalized to the wild-type strain SHY169. **b** SDS-PAGE analysis of secreted cellulases in the supernatants of the selected mutants. **c** Comparison of fermentation performance of the best strain R5-2 and the wild-type in 5-L fermenter. Data are the average mean of triplicate samples, and the error bars represent standard deviations. Wet cell weight was measured as follows. Ten mL fermentation broth sample was centrifuged at 8000 g for 10 min in pre-weighed tube. The supernatant was removed. The wet weight of the pellet was measured and calculated to the unit g/L

Secondary validation results showed that more than one third of mutants seemed to rapidly degenerate (Additional file 1: Fig. S2), which consisted with the previously reported genome instability of mutagenized *P. pastoris* [15, 47]. Notably, some mutants from the third and fourth rounds, whose primary validation rates were relatively high (Fig. 5a), such as R3–4, R4–5, and R4–12, maintained the significance of improved performance (Additional file 1: Fig. S2). Thus, we selected one best producers from the first and second rounds, top three producers from the third and fourth rounds as well as top two producers for final validation. Eventually, compared to the wild-type stain SHY169, most of the selected mutants except for R4–6 exhibited significantly increased cellulase production by 1.5–2.1-fold as well as yield by 1.3–1.8-fold (Fig. 6a). The supernatants from cultures of these strains were analyzed by SDS-PAGE and protein concentration measurements, which confirmed the improved secretion of cellulase by the mutants (Fig. 6b and Additional file 1: Fig. S3). Among them, the mutant stain R5–2, which was obtained from the fifth round, showed the best cellulase production performance with a 2.1(± 0.1)-fold increase of production and a 1.8(± 0.1)-fold increase of yield (Fig. 6a). High-density fermentation experiments in a 5-L bioreactor were conducted to further compare cellulase production capacities between the mutant strain R5–2 and the wild-type strain SHY169. The fermentation profiles showed that the mutant R5–2 had a faster and higher cell growth than the wild-type SHY169 (Fig. 6c), where the final cell growth of R5–2 increased 1.3-fold than that of SHY169. Correspondingly, R5–2 exhibited faster and higher cellulase production than that of SHY169, where R5–2 produced a 1.9-fold increase of cellulase (11,110 ± 523 U/mL) by a 1.7-fold increase of rate (74.6 U/mL/h) compared to SHY169 (Fig. 6c).

### Elucidation of genomic variants in mutant strains with enhanced cellulase production

To uncover genomic alterations accumulated during iterative ARTP mutagenesis, the ten selected mutant strains according to the final validation results (Fig. 6a) and the wild-type strain were subjected to whole-genome resequencing. By comparing with the reference genome of strain GS115, different SNPs and InDels were detected in the mutant strains versus the starting strain in each round (Additional file 2: Dataset S1). Here, only mutations in coding regions were considered. Total three SNPs (positions: 2290916 and 214209 on Chromosome 2, 1516266 on Chromosome 4) were found in coding regions, thereby causing nonsynonymous amino acid changes in three proteins, including Rsc1 (G22V, glycine to valine at position 22), Rvs167 (G9R, glycine to arginine at position 9) and Pmt6 (V57M, valine to methionine at position 57). Rsc1 (GeneBank accession No. XP_002492164) is a component of the RSC chromatin remodeling complex [48], and its homolog in *Saccharomyces cerevisiae* was reported to be involved in general transcription regulation [49]. Rvs167 (GeneBank accession No. XP_002491000) is an actin-associated protein, and its homolog in *S. cerevisiae* was reported to play roles in endocytosis and exocytosis as well as interact with proteins involved in ER to Golgi vesicle trafficking [50, 51]. Pmt6 is one of protein-O-mannosyltransferases, which was previously characterized to play a minor role in O-glycosylation in *P. pastoris* [52].

**Fig. 7:**
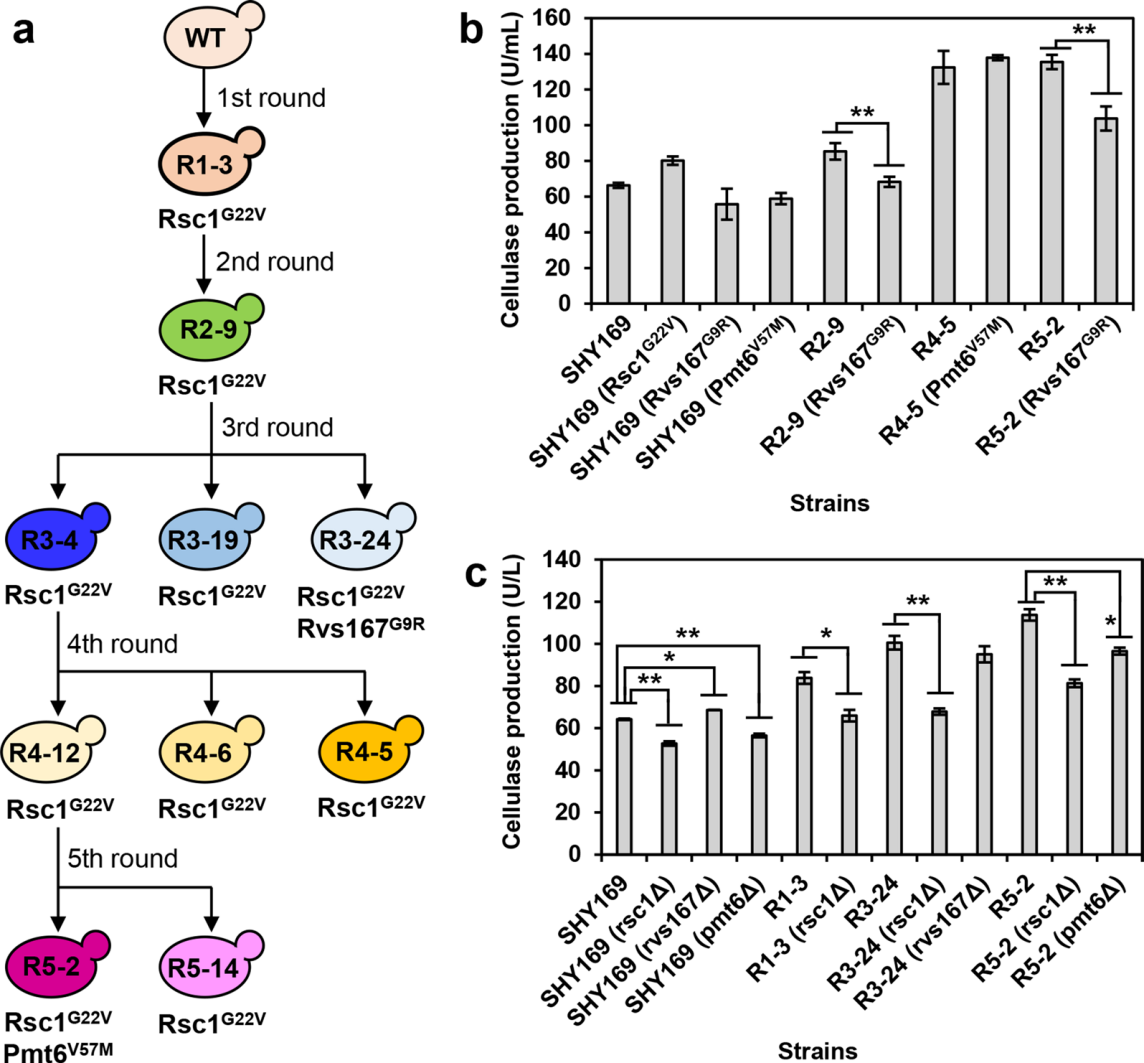
Identification of genomic variants using genome resequencing and genetic validation methods. **a** Protein point mutations uncovered by genome resequencing to be accumulated during the iterative ARTP mutagenesis and microfluidic screening. **b** Cellulase production in wild-type and reconstructed point mutation strains. **c** Cellulase production in wild-type and key gene deletion strains. Data represent the mean and standard error of duplicate cultures for each strain. Statistical analysis for each group of strains including the starting strain (SHY169, R1–3, R3–24, or R5–2) and its derived deletion strains was performed using one-way ANOVA followed by Tukey’s multiple-comparison posttest (**P < 0.01, *P < 0.05)

To further trace their accumulations during iterative mutagenesis, these three mutations were mapped onto the mutagenesis lineage (Fig. 7a). The mutation of Rsc1^G22V^ was observed to be generated in the first round of ARTP mutagenesis and passed on to all the next generations. The stable inheritance of Rsc1^G22V^ in high cellulase-producing mutants implicated that Rsc1 might play an important role in cellulase production. In addition, besides Rsc1^G22V^, Rvs167^G9R^ was found in the mutant R3–24 selected from the third round, which showed the lowest cellulase production among the three selected strains (Fig. 6a). Thus, it seemed that Rvs167 might have a minor or even negative effect on cellulase production. Interestingly, besides Rsc1^G22V^, the best cellulase producer R5–2 selected from the fifth round was found to accumulate the other mutation Pmt6^V57M^, suggesting its additive beneficial effect on cellulase production.

To validate the effects of Rsc1^G22V^, Rvs167^G9R^, and Pmt6^V57M^ on cellulase production, point mutations were generated in appreciate strains using CRISPR/Cas9 system. As shown in Fig. 7b, introduction of Rsc1^G22V^ in the wild-type strain SHY169 resulted in 1.2-fold higher cellulase production than the control stain SHY169. This result confirmed the positive and dominant roles of Rsc1^G22V^, which was accumulated in all the selected mutant strains (Fig. 7a), in cellulase production (Fig. 6a). Introductions of Rvs167^G9R^ in the wild-type strain as well as the selected mutant strains R2–9 and R5–2 resulted in 0.84-, 0.80-, and 0.76-fold lower cellulase production than their corresponding control strains (Fig. 7b), thus confirming the negative effect of Rvs167^G9R^ on cellulase production. Introduction of Pmt6^V57M^ in the selected mutant strain R4–5 resulted in 1.04-fold higher cellulase production than the control strain R4–5 (Fig. 7a), confirming the slightly positive effect of Pmt6^V57M^ on cellulase production.

Next, gene deletion method was employed to further validate the effects of Rsc1, Rvs167, and Pmt6 on cellulase production (Fig. 7c). All the selected mutant strains were found to have the point mutation in *RSC1* by genome sequencing (Fig. 7a). Deletion of *RSC1*, either in the wild-type strain SHY169 or some mutant strains, such as R1–3 from the first round, R3–24 from the third round, and R5–2 from the fifth round, resulted in significantly decreased cellulase production. This result suggested that *RSC1* might play an important role in cellulase production. Deletion of *RVS167* resulted in significantly increased cellulase production in the wild-type strain SHY169, but not in the mutant strain R3–24 harboring the mutant Rvs167^G9R^. This result implicated that *RVS167* might play a negative role in cellulase production, but relatively minor. *PMT6* point mutation was found in the mutant strain R5–2. Deletion of *PMT6* resulted in a significantly 0.85-fold decrease in cellulase production by R5–2, but its impact was slightly lower than deletion of *RSC1*, which showed a 0.71-fold decrease. By contrast, the effects of *PMT6* and *RSC1* on cellulase production in the wild-type strain SHY169 were similar, where 0.88-fold and 0.82-fold decreases were observed, respectively. These results suggested that *PMT6* might play a positive but minor role in cellulase production.

Overall, point mutation and gene deletion experiments further confirmed the potential functions of Rsc1, Rvs167, and Pmt6, which accumulated point mutations during iterative ARTP mutagenesis, in cellulase production.

## Discussion

*P. pastoris* is one of the most attractive hosts for the heterogenous and large-scale production of cellulases. Due to limited understanding of complicated cellular metabolism, whole-cell directed evolution has played a significant role in strain improvement to increase enzyme production. However, the inefficient of conventional plate-based screening is a major bottleneck. In the past decade, droplet microfluidics has emerged as a powerful tool for high-throughput screening. A previous study reported that a gel microdroplet-based high-throughput screening method was employed to screen *P. pastoris* strains with improved xylanase production at a throughput of up to 10^8^ variants per day [53]. Compare to gel microdroplet, the generation and manipulation (including splitting, fusion, trapping, injection of reagent, and sorting) of water-in-oil droplet were more flexible [54]. Here, we present a water-in-oil droplet microfluidic method for high-throughput screening of mutant *P. pastoris* strain libraries for improved cellulase production.

Compared to flow cytometry-based high-throughput screening, an important advantage of droplet-based screening method is that each droplet serving as an individual microreactor to link genotype (the enzyme-encoding gene) and phenotype (the reaction product) through compartmentalization. This linkage relies on the integrity of the droplet. Therefore, the droplet stability and fluorescein leakage were tested in the presence of methanol in the aqueous phase, and the results showed that after 48 h off-chip incubation, the droplets remained stable and monodisperse in the methanol concentration range of 0.5–2%, and the fluorescein leakage was insignificant in the presence of 1% methanol in droplets (Additional file 1: Fig. S1). Other factors that impact droplet integrity have previously been reported and should be noted, including the choice of oil-surfactant pairs, droplet incubation conditions, and the addition of Bovine Serum Albumin prevents leaking, etc. [38].

The droplet microfluidic screening platform provides higher throughputs and lower reagent consumption compared to traditional microplate-based screening. In this study, *P. pastoris* single cells were encapsulated in 10 pL droplets at a rate of 3000 droplets per second, and droplets were sorted at 300 droplets per second based on their fluorescence intensity (Fig. 4). The total reagent consumption was less than 2 mL in each round of screening. After five sequential rounds of mutagenesis and selection, several mutants with improved cellulase production were successfully isolated and the best-performing clone exhibited a more than twofold increase in cellulase production (Fig. 6). Furthermore, the microfluidic screening system could potentially be adapted for engineering and screening strains for the expression of other enzymes or even other metabolites as long as they can be linked to a fluorescent signal.

In addition, with the rapid development of high-throughput sequencing technology and decrease in sequencing costs, whole-genome sequencing technology has now enabled us to analyze genotype–phenotype correlations of mutants with improved performance, identify the genetic changes and mechanisms for the altered phenotype, and combine with accurate genetic modification techniques, to rationally guide and optimize strain improvement. Huang et al. reported used droplet microfluidics screening of whole-genome yeast mutant libraries for improved α-amylase secretion, and performed whole-genome sequencing of several isolated strains, and identified 330 mutations that may contribute to improved protein secretion [35]. Balasubramanian et al. reported used random mutagenesis and droplet-based microfluidic to successfully improve β-glucosidase secretion in *C. glutamicum*, and whole-genome resequencing and functional enrichment analysis found mutations involved in protein synthesis and secretion relevant biological processes [55]. Similar to previous studies, we also sequenced the whole-genomes of the improved cellulase production mutants from each library together with the original strain, several mutations that may contribute to the improved cellulase secretion were identified (Fig. 7). These mutations identified in this study can potentially be transferred to other platform strains by reverse metabolic engineering for the production of other proteins. The combined use of microfluidics screening and whole-genome sequencing could broadly facilitate the design of novel cell factories.

## Conclusions

In summary, we developed a droplet-based microfluidic platform for high throughput screening of *P. pastoris* strains with improved cellulase production at a throughput of up to 300 drops per second. We performed five rounds of whole-genome mutagenesis and selection and obtained a *P. pastoris* mutant strain with about twofold increase in cellulase production. Mutated genes identified by whole-genome sequencing were demonstrated that affecting heterogenous protein expression. Furthermore, the droplet-based microfluidic HTS platform might be also applicable to accelerate the whole-cell directed evolution of host strains of other secreted enzymes.

## Methods

### Strains, media, and culture conditions

The strains used in this study are listed in Additional file 1: Table S1. *P. pastoris* SHY169 derived from GS115 (Mut^+^, Additional file 1: Table S1), in which a codon-optimized endo-β-D-1,4-glucanase gene *stce1* from *Staphylotrichum coccosporum* was integrated by single copy, was provided by Wuhan Sunhy Biology Co., Ltd (Wuhan, China) and used as the starting strain for ARTP mutagenesis. The nucleotide sequence alignment between the wild-type and codon-optimized *stce1* genes is shown in Additional file 1: Fig. S4.

To validate the effects of point mutations and mutant genes found in ARTP mutant strains on cellulase production, both reconstructed point mutation strains and gene deletion strains were generated. The PCR primers used in this study are listed in Additional file 1: Table S2. Point mutations were reconstructed using CRISPR-Cas system [56]. One Step Cloning kit (Vazyme Biotech, Nanjing, China) was employed to construct plasmids containing Cas9 and specific gRNA. First, a template hygromycin-resistant plasmid containing the Cas9 and the gRNA expression cassette, which can be used in *P. pastoris* strains in this study, was construct. The plasmid backbone was PCR amplified from the vector pPpT4-pHTX1-hsCas9-GUT1-gRNA2 to get the linearized fragment. The hphB fragment was obtained from the template vector pRS426-hphB [57]. The resulting plasmid was named pCas9-gRNA-hphB. N20 gRNA sequences were designed to target genes of interest and inserted into the linearized pCas9-gRNA-hphB plasmid, resulting in the target-specific CRISPR-Cas9 plasmids. Homologous recombination (HR) donor cassettes were amplified from genomic DNA of point mutation strains using primer pairs listed in Additional file 1: Table S2. One μg linear HR donor cassette and 200 ng target-specific CRISPR-Cas9 plasmid were used for yeast transformation. Gene deletion strains were generated using PCR-mediated homologous recombination method [58]. Hygromycin-resistance gene was PCR amplified from the plasmid pRS426-hphB, and its expression was controlled under *S. cerevisiae TEF1* promoter and terminator [57]. The PCR fragments containing hygromycin-resistance expression cassettes flanked by 1000-bp homologous sequences to the target genes were obtained using fusion PCR. The 1000-bp homologous sequences upstream and downstream of the target genes were PCR amplified from SHY169 genomic DNA. Yeast transformation was performed using the electrotransformation method [59]. Positive transformants were selected on hygromycin selective plates, which were YPD agar plates containing 200 μg/ml hygromycin B. Successful point mutations were verified using PCR amplification of the target locus and Sanger sequencing (for primers, see Additional file 1: Table S2). Successful gene deletions were verified by diagnostic PCR reactions with primers designed on the target genes as well as hygromycin-resistance cassette (for primers, see Additional file 1: Table S2).

The YPD medium contained, per liter, 10 g yeast extract, 20 g peptone, and 20 g glucose. The BMGY liquid medium contained, per liter, 10 g yeast extract, 20 g peptone, 2.3 g K_2_HPO_4_, 11.8 g KH_2_PO_4_, pH 6.0, 13.4 g yeast nitrogen base (YNB), 0.4 mg biotin, and 10 ml glycerol. The BMMY medium contained as per BMGY except with the indicated amount of methanol instead of the glycerol. The basal fermentation medium for 5-L jar fermenter included, per liter, 27 g NH_4_H_2_PO_4_, 3.7 g KH_2_PO_4_, 13.2 g MgSO_4_, 13.5 g K_2_SO_4_, 0.73 g CaSO_4_, 1.06 g KOH, 0.44 g Biotin, and 40 g Glycerol. Unless stated otherwise, cells were cultured in YPD or BMGY media at 30 °C, 250 rpm. Cells for inducing cellulase production were grown in BMMY medium at 28 °C, 250 rpm. Fed-fermentation was conducted as follows. Cells were cultured in 60 ml YPD at 28 °C, 150 rpm in 500 ml baffled shaker flasks for 48 h, then transferred to the 5-L jar fermenter containing 2 L of the basal fermentation medium. Methanol was supplemented according to the fermentation process to ensure that methanol concentration was maintained at 0.5 to 1% (v/v).

### Cellulase assay

To establish a method of monitoring the cellulase activity in droplets, a fluorogenic substrate fluorescein di-beta-D-cellobioside (FCB, AAT bioquest), was used [46]. Under cellulase treatment, FCB releases the green-fluorescent compound fluorescein, whose intensity can be titrated with fluorescence detection (Ex 473 nm/Em 520 nm). To verify the detection range of this fluorescence-based cellulase activity measurement method, activity of commercial cellulase HT639plus (a commercial product produced by SHY169 strain, Wuhan SunHY Biological Co., Ltd) was assayed in the microfluidic droplets [21], and the microtiter plate (MTP)-based assay format was used as control. Reactions contained 20 μM FCB and the indicated amounts of cellulase (1–4 U/mL), which were dissolved in BMMY medium with 1% methanol to mimic the actual droplet screening. The reaction mixture was rapidly encapsulated into the droplets or pipetted into microplate wells, respectively. After incubating at 30 °C for 48 h, the fluorescence was measured using the custom droplet screening device in our lab for the droplet-based assay format and using the SpectraMax M2e Microplate Reader (Molecular Devices Corporation) for the MTP-based assay format, respectively.

A traditional approach of measuring cellulase activities using the 3,5-dinitrosalicylic acid (DNS) reduction method was also used in this study, in which carboxymethyl cellulose sodium (CMC-Na, from Sigma-Aldrich) was used as the substrate [5]. In a reaction, 0.2 mL of appropriately diluted culture supernatant was added to 1.8 mL substrate solution containing 1.5% (w/v) CMC-Na in 100 mM phosphate buffer (87.7 mM NaH_2_PO_4_, 12.3 mM Na_2_HPO4, pH 6.0). The reactions were incubated at 50 ˚C for 10 min, stopped by the addition of 3 mL DNS reagent, followed by 5 min boiling. After cooling down to room temperature, the optical density of the solution was measured at 540 nm, and the concentration of reducing sugars was estimated using a glucose standard curve as reference. One unit of the enzyme activity was defined as the amount of enzyme that released 1 μmol of glucose per minute.

### Droplet‑based microfluidics

#### Fabrication of microfluidic chips

Microfluidic chips were fabricated in poly(dimethylsiloxane) and glass using standard soft lithographic methods [39]. The chips were used for droplet generation and sorting, respectively. The chips had channel heights about 25 μm for droplet-making or sorting, and 0.5-mm diameter holes for channel inlets and outlets. For the sorting chip, microelectrodes were fabricated by heating the chip to 95 °C and pushing low-melting-temperature solder wire into the electrode channels. A hydrophobic surface treatment was applied to the microfluidic channel walls by injecting Aquapel and then flushing with pressurized air.

#### Optical setup, data acquisition and control system

The optical setup for droplet fluorescence detection consisted of a 100 mW, 473 nm high stability blue laser and a Nikon TI-U inverted microscope. The laser beam was passed through a beam expander, then guided into the backport of the microscope. The laser light inside the microscope was reflected by a dichroic beam splitter (DBS) and then focused on the sorting chip through the objective lens. The fluorescent emission from each droplet was passed back along the path of the laser and separated by the DBS to the photomultiplier tube (PMT, Hamamatsu Photonics), which was mounted to the microscope’s side port. A high-speed video camera (Fastec HiSpec 1, Fastec Imaging, San Diego, CA, USA) was mounted on the microscope for the imaging of droplet formation, reinjection, and sorting. Data acquisition (DAQ) and control were conducted with a DAQ card (National Instruments) executing a program written in LabVIEW software (National Instruments). When the droplet fluorescence intensity exceeds a defined threshold, the DAQ card provided a signal to a high voltage amplifier (TREK 609E-6), which is connected to the electrodes of the sorting chip, to sort the corresponding droplet.

#### Droplet generation and microfluidic screening

Monodispersed droplets were generated using a droplet-making chip by flow-focusing the aqueous phase with two streams of fluorinated oil (HFE-7500, 3 M) containing 2% (wt/wt) surfactant (Raindance Technologies) [60]. The aqueous and oil phases were loaded into 1-ml disposable syringes (BD Biosciences), respectively, and pumped into the droplet-making chip using the syringe pumps (Harvard Apparatus Inc.). Syringes were connected to the droplet-making chip using PTFE tubing with an internal diameter of 0.38 mm and an external diameter of 0.76 mm. The syringe pumps were operated with the aqueous flow rates of 100 μL/h and the oil flow rates of 300 μL/h, to produce 10-pL droplets at a rate of 3000 droplets per second. The resulting droplets were collected into a 1-mL syringe and incubated for the desired time at 30 °C. After incubation, the droplets were reinjected into a microfluidic sorting chip at a flow rate of 10 μL/h and spaced by HFE-7500 oil without surfactant infused at 200 μL/h. The emitted fluorescence signal of each droplet was detected by the optical setup. When the droplet fluorescence intensity exceeded a defined threshold, the electrodes were activated, and the droplets were subsequently sorted to the sorting channel, whereas unwanted droplets flowed into the waste channel. The sorted droplets were collected into an Eppendorf tube for further experiments.

#### Assessment of droplet stability and fluorescence leakage

To test the effects of methanol on the droplet stability and fluorescence leakage, the BMMY medium containing methanol was tested as the aqueous phase for generating droplets. For testing the droplet stability, the droplets containing different concentrations of methanol (0, 0.5%, 1%, or 2%; v/v) were collected into the syringes and incubated at 30 °C for 48 h. Droplets were imaged using Lecia DM5000B microscope (Lecia Microsystems) and compared at the time of droplet formation and during the incubation. The droplet sizes were measured by ImageJ analysis as previously described [61]. To determine fluorescence leakage, three types of droplets, including fluorescent and nonfluorescent droplets as well as their binary mixture, were generated and collected into syringes, respectively. Fluorescent droplets were generated from a 40 h reaction solution of cellulase in BMMY medium with 1% methanol and 20 μM fluorogenic substrate FCB. Nonfluorescent droplets only contained BMMY medium with 1% methanol. The binary mixture contained 10 μL of fluorescent droplets and 100 μL of nonfluorescent droplets. All the droplets in syringes were incubated at 30 °C for 48 h, and separately reinjected into the sorting chip. And then, their fluorescence distributions were counted.

#### Optimal droplet incubation time and verification of the droplet-based microfluidic platform

The optimal droplet incubation time was determined by growing the cellulase-producing strain SHY169 in droplets, which were detected for green fluorescence signals by imaging with Lecia DM5000B fluorescence microscope (Lecia Microsystems) every 8 h for 48 h. The droplet-based microfluidic platform was verified by measuring the sorting efficiency of an artificial cell library, which consisted of cellulase-producing and non-cellulase-producing strains. Cellulase-producing strain SHY169 and non-cellulase-producing strain GS115 were grown in BMGY medium overnight at 30 °C, 250 rpm. Cells were pelleted by centrifuging at 3000 × g for 2 min, washed twice and resuspended in BMMY medium, and sonicated for 3 × 10 s at 40 W to avoid cell adhesion. Cell suspensions of SHY169 and GS115 were mixed at a 1:99 ratio, and then diluted to a concentration of 2 × 10^7^ cells per ml in BMMY medium containing 20 μM FCB and 10% (v/v) density-matching solution (OptiPrep). The mixture was encapsulated in droplets at a cell to droplet ratio of 0.2 according to the law of Poisson distribution, and incubated off-chip at 28 °C for 40 h. The droplets were reinjected into the sorting chip, and sorted according to their fluorescence intensities at a rate of 300 droplets per second. Cells were recovered from the sorted droplets by adding 50 μL of 1H,1H,2H,2H-perfluorooctanol and gently vertexing to break the droplets, and spread on YPD agar plates to allow growth for 2 days. Ninety colonies were randomly picked and used for cellulase activity measurement using the DNS reduction method [5].

### ARTP mutagenesis and microfluidic screening

ARTP was employed to iteratively mutagenize cellulase-producing strain SHY169. A single colony from a fresh plate was inoculated into YPD medium and grown overnight at 30 °C, 250 rpm. Cells were pelleted and washed twice with sterile saline, and diluted to OD_600_ = 1 (correspond to 1 × 10^8^ cells per ml). Subsequently, 10 μL cell suspension was spread on a sterilized stainless-steel plate and subjected to ARTP treatment for 30 s. The operating parameters were as follows, including a radio frequency power input of 100 W, a helium gas flow rate of 10 SLM (standard liters per minute), and a 2 mm distance between the plasma torch nozzle exit and the sample plate. The ARTP mutagenesis procedure was repeated five times. All cells from the five ARTP treated plates were collected in a new sterile tube containing 450 μL of YPD medium, and spread on YPD agar plates. The agar plates were incubated at 30 °C until colonies formed.

Cells were scraped from the agar plates, washed, and resuspended in fresh BMMY medium. Cell suspension was sonicated for 3 × 10 s at 40 W and filtered using an 8-μm filter (Whatman) to avoid cell adhesion. OD_600_ was measured, and cell suspension was diluted to 2 × 10^7^ cells per ml to achieve a cell-to-droplet ratio of 0.2. The diluted cell suspension was subsequently encapsulated into droplets together with 20 μM fluorogenic substrate FCB. The generated droplets were collected into a 1-ml syringe preloaded with 100 μL HFE-7500 oil, and then incubated at 30 °C for 40 h. Next, the droplets were sorted based on the resulting fluorescence using the sorting chip. The sorted droplets were collected in a sterile Eppendorf tube with 200 μL fresh YPD medium. Cells were recovered from the sorted droplets, spread on YPD agar plates, and incubated at 30 °C until colonies formed. About thirty colonies were randomly picked and used for further validating cellulase production by primary validation of flask fermentation, and then the isolates with the top five high performance were applied to secondary validation. The mutant strain with the stable and highest cellulase production was subjected to the next round of ARTP mutagenesis and microfluidic screening.

### Characterisation of cellulase production by selected mutants

Cellulase production by selected mutants was characterized by flask fermentation and 5-L bioreactor as specified in the text. The selected colonies from every round of ARTP mutagenesis and microfluidic screening were inoculated in 2 ml BMGY medium, and grown to the logarithmic period at 30 °C, 250 rpm. Cells were harvested by centrifuging at 3000 × g for 5 min at room temperature and resuspended to an OD_600_ of 1.0 in 10 ml BMMY medium containing 1% methanol in a 50-mL flask. After incubation for 40 h at 28 °C, 250 rpm to induce cellulase production, OD_600_ of each fermentation broth was measured, and cellulase activity of fermentation supernatant was analyzed using the DNS reduction method. Finally, the top performer from flask fermentation was further analyzed for cellulase production in 5-L batch fermentation using the starting strain SHY169 as a control.

### Protein analysis

Secreted cellulase in culture supernatants was analyzed by sodium dodecyl sulphate–polyacrylamide gel electrophoresis (SDS-PAGE). The supernatants were mixed with a 5 × SDS-PAGE sample buffer (0.5 M Tris–HCl pH 6.8, 10% SDS, 50% glycerol, 5% β-mercaptoethanol, 0.05% bromophenol blue), and boiled for 5 min. Then 20 µL of samples were loaded on gradient (8–16%) precast polyacrylamide gel (SurePAGE, GenScript Biotech Corp., Nanjing, China) and stained with Coomassie blue after gel electrophoresis at 120 V for 40 min. The protein concentration was determined using Quick Start™ Bradford protein assay kit (Bio-Rad Laboratories).

### Genome resequencing and data analysis

The wild-type strain SHY169 and ten selected mutant strains with enhanced cellulase production were grown in 200 ml liquid YPD media at 30 °C to exponential phase. Cells were harvested by centrifuging for 5 min at 4000 × g. The supernatants were discarded, and the pellets were stored at − 80 °C until further use for genome resequencing. Genomic DNA isolation and the sequencing libraries were constructed and sequenced on Illumina HiSeq Xten-PE150 using 150-bp paired-end sequencing by Novogene (Beijing, China). A mean of 9.2 million 150-bp clean reads was generated for each library. The genome sequencing raw data were deposited in the NCBI Sequence Read Archive under the accession number PRJNA772571. Sequence analysis and variant detection were performed as previously described [62]. The *P. pastoris* GS115 genome as a reference was downloaded from RefSeq at the NCBI (sequence assembly version ASM174695v1, RefSeq assembly accession: GCA_001746955.1). The Genome Analysis Toolkit (GATK v3.5) Best Practices pipeline was employed to detect single nucleotide polymorphisms (SNPs) and insertion/deletion (InDels) [63, 64]. The cleaned reads were mapped to the reference genome using the mapping tools BWA-mem (version 0.7.13) [65]. Variant callings were performed using GATK HaplotypeCaller. Variant annotation was conducted using the package ANNOVAR [66]. The genomic variants including SNPs and InDels as well as the protein-altering variants in comparisons between the starting strains and the subsequent selected strain during the iterative ARTP mutagenesis and microfluidic screening are available in supplementary material (Additional file 2: Dataset S1; Additional file 3: Dataset S2).

## Supplementary Information

**Additional file 1: Table S1**. P. pastoris strains used in this study. Table S2. Primer used in this study. **Fig. S1**. Effects of methanol concentration on droplet stability. a Mean diameter change of droplets containing different amounts of methanol. Droplets were off-chip incubated for 48 h at 30 °C. Data are presented as means ± standard deviations of three independent experiments. b Image of droplets containing 2% methanol at 48 h. Scale bar: 50 μm. **Fig. S2**. Secondary validation of selected mutants by flask fermentation. Top five cellulase hyperproducers from each round of five iterative ARTP mutagenesis and screening were evaluated for cellulase production by flask fermentation. Production is measured in units of cellulase activity per volume and the yield is cellulase activity per 1 OD600 unit of cells. Data were normalized to the starting strain of each round. The verified strains were indicated by red stars, and used for the final validation and genome resequencing analysis. **Fig. S3**. Extracellular protein concentrations of the selected mutants. **Fig. S4**. Nucleotide sequence alignment between the wild-type (WT) and codon-optimized (CO) Staphylotrichum coccosporum stce1 genes. The stce1 wild-type sequence was from GenBank accession no. AB248917.1.

**Additional file 2: Dataset S1**. Genomic variants in mutant strains obtained from five rounds of ARTP mutagenesis of cellulase-producing *P. pastoris*.

**Additional file 3: Dataset S2**. Genomic variants in coding regions.

## Data Availability

All data generated or analyzed during this study are included in this published article and its additional files.
