# Supplementary material for: Microfluidic screening and genomic mutation identification for enhancing cellulase production in Pichia pastoris
Source: Biotechnol Biofuels Bioprod. 2022 May 14;15:50. doi: 10.1186/s13068-022-02150-w (PMC9107654; doi:10.1186/s13068-022-02150-w)
Supplement: Supplementary file 1 — Additional file 1: Table S1. P. pastoris strains used in this study. Table S2. Primer used in this study. Fig. S1. Effects of methanol concentration on droplet stability. a Mean diameter change of droplets containing different amounts of methanol. Droplets were off-chip incubated for 48 h at 30 °C. Data are presented as means ± standard deviations of three independent experiments. b Image of droplets containing 2% methanol at 48 h. Scale bar: 50 μm. Fig. S2. Secondary validation of selected mutants by flask fermentation. Top five cellulase hyperproducers from each round of five iterative ARTP mutagenesis and screening were evaluated for cellulase production by flask fermentation. Production is measured in units of cellulase activity per volume and the yield is cellulase activity per 1 OD600 unit of cells. Data were normalized to the starting strain of each round. The verified strains were indicated by red stars, and used for the final validation and genome resequencing analysis. Fig. S3. Extracellular protein concentrations of the selected mutants. Fig. S4. Nucleotide sequence alignment between the wild-type (WT) and codon-optimized (CO) Staphylotrichum coccosporum stce1 genes. The stce1 wild-type sequence was from GenBank accession no. AB248917.1. [file 13068_2022_2150_MOESM1_ESM.pdf]

Supporting Information

Table S1. *P. pastoris* strains used in this study

Strain	Description	Reference or source
GS115	*P. pastoris* strain, Mut^+	Invitrogen, USA
SHY169	*P. pastoris* strain with integrated cellulase gene (a codon-optimized endo-β-D-1,4-glucanase gene stce1 from *Staphylotrichum coccosporum*) in the genome, derived from GS115, Mut^+	Wuhan Sunhy Biology Co., Ltd
R1-3	mutant strain from the first round of ARTP mutagenesis of *P. pastoris* SHY169 and directly derived from the strain SHY169	This study
R2-9	mutant strain from the second round of ARTP mutagenesis of *P. pastoris* SHY169 and directly derived from the strain R1-3	This study
R3-4	mutant strain from the third round of ARTP mutagenesis of *P. pastoris* SHY169 and directly derived from the strain R2-9	This study
R3-19	mutant strain from the third round of ARTP mutagenesis of *P. pastoris* SHY169 and directly derived from the strain R2-9	This study
R3-24	mutant strain from the third round of ARTP mutagenesis of *P. pastoris* SHY169 and directly derived from the strain R2-9	This study
R4-5	mutant strain from the fourth round of ARTP mutagenesis of *P. pastoris* SHY169 and directly derived from the strain R3-4	This study
R4-6	mutant strain from the fourth round of ARTP mutagenesis of *P. pastoris* SHY169 and directly derived from the strain R3-4	This study
R4-12	mutant strain from the fourth round of ARTP mutagenesis of *P. pastoris* SHY169 and directly derived from the strain R3-4	This study
R5-2	mutant strain from the fifth round of ARTP mutagenesis of *P. pastoris* SHY169 and directly derived from the strain R4-6	This study
R5-14	mutant strain from the fifth round of ARTP mutagenesis of *P. pastoris* SHY169 and directly derived from the strain R4-6	This study
SHY169 (rsc1Δ)	SHY169 rsc1 deletion strain, rsc1Δ::hphB	This study
SHY169 (rvs167Δ)	SHY169 rvs167 deletion strain, rvs167Δ::hphB	This study
SHY169 (pmt6Δ)	SHY169 pmt6 deletion strain, pmt6Δ::hphB	This study
R1-3 (rsc1Δ)	R1-3 rsc1 deletion strain, rsc1Δ::hphB	This study
R3-24 (rvs167Δ)	R3-24 rvs167 deletion strain, rvs167Δ::hphB	This study
R5-2 (pmt6Δ)	R5-2 pmt6 deletion strain, pmt6Δ::hphB	This study

Table S2. Primer used in this study

Name	Sequence (5' → 3') Application	Application
HyhB-F	CACATCCGAACATAAACAACCATGAAAAAGCCTGAACTCACCG	Amplify hphB gene
HyhB-R	CTTTTTATTGTCAGTACTGACTATTCCTTTGCCCTCGGACG	Amplify hphB gene, reverse primer for verifying gene deletion using hphB expression cassette
Ptef-F	GACATGGAGGCCCAGAATACC	Amplify *S. cerevisiae* TEF1 promoter
Ptef-R	GGTTGTTTATGTTCGGATGTGATG	Amplify *S. cerevisiae* TEF1 promoter
Ttef-F	TCAGTACTGACAATAAAAAGATTCTTG	Amplify *S. cerevisiae* TEF1 terminator
Ttef-R	CAGTATAGCGACCAGCATTCACATAC	Amplify *S. cerevisiae* TEF1 terminator
RSC-HR1-F	CATACAGATTGACTGGTGCCGATAAC	Amplify 1 kb homologous sequence upstream *RSC1*
RSC-HR1-R	GTATTCTGGGCCTCCATGTCCAGAGATCAGCGTATTTCGTGTTTG	Amplify 1 kb homologous sequence upstream *RSC1*
RSC-HR2-F	GAATGCTGGTCGCTATACTGATAGGTCGATTCTTGTATATTGTAACAAT	Amplify 1 kb homologous sequence downstream *RSC1*
RSC-HR2-R	AAGTTACGATACGAAGAATTGAGAAGAAG	Amplify 1 kb homologous sequence downstream *RSC1*
RSC1-F	GTCGGAGGCTTTCAAGGAAG	Verify *RSC1* deletion
RSC1-R	GATTCTTACCTTCTGTAGGGACATCG	Verify *RSC1* deletion, negative control
RVS-HR1-F	CACGCTTGTATTTATGTCTGTACAAAGG	Amplify 1 kb homologous sequence upstream *RVS167*
RVS-HR1-R	GTATTCTGGGCCTCCATGTCATCGGATAGTTCGCAATTGAGG	Amplify 1 kb homologous sequence upstream *RVS167*
RVS-HR2-F	GAATGCTGGTCGCTATACTGTGCTAAATGTCAAATATCAAGAATCTATACG	Amplify 1 kb homologous sequence downstream *RVS167*
RVS-HR2-R	ACAATTAATGATCTCATGTCTGACGTG	Amplify 1 kb homologous sequence downstream *RVS167*
RVS167-F	CAGAGGAATAAGTGGCTCTGATAGTG	Verify *RVS167* deletion
RVS167-R	CTGAATCCAATTTGATGGTTCAAC	Verify *RVS167* deletion, negative control
PMT-HR1-F	CACTTGATTATCTAGGAAAGGCATTATC	Amplify 1 kb homologous sequence upstream *PMT6*
PMT-HR1-R	GTATTCTGGGCCTCCATGTCCTCGGATTGTAGAGAACAATTTGTC	Amplify 1 kb homologous sequence upstream *PMT6*
PMT-HR2-F	GAATGCTGGTCGCTATACTGGGAAGAAGGTGTAGGGCAAATATTAG	Amplify 1 kb homologous sequence downstream *PMT6*
PMT-HR2-R	GCAAAGCTATTAGAAAAATTCTGTAGC	Amplify 1 kb homologous sequence downstream *PMT6*
PMT6-F	CGTCTCTAACCAGACTGATTACTTCG	Verify *PMT6* deletion
PMT6-R	CAGCATTGAATATCCTCATTACCTTG	Verify *PMT6* deletion, negative control
Pcas-F	CAATTGACACCTTACGATTATTTAGAGAG	For constructing the plasmid pCas9-gRNA-hphB
Pcas-R	GGTTTAGTCCTCCTTACACCTTGTC	For constructing the plasmid pCas9-gRNA-hphB
phygBF	GACAAGGTGTAAGGAGGACTAAACCATGAAAAAGCCTGAACTCACCG	For constructing the plasmid pCas9-gRNA-hphB
phygBR	CTCTCTAAATAATCGTAAGGTGTCAATTGCTATTCCTTTGCCCTCGGAC	For constructing the plasmid pCas9-gRNA-hphB
seqhygcas-F	GAGCACTTCATTGTGTTGCGC	Sequencing primer of the plasmid pCas9-gRNA-hphB
Seqhygcas-R	GCAACGTGACACCCTGTGCAC	Sequencing primer of the plasmid pCas9-gRNA-hphB
Rsc1gRF	ACGAAACGAGTAAGCTCGTCACGTAGACATTATCTACCAAGTTTTAGAGCTAGAAATAGCAAGTT	For constructing *RSC1* specific Cas9-gRNA plasmid
Rsc1gRR	GACGAGCTTACTCGTTTCGTCCTCACGGACTCATCAGACGTAGTTTGATTTGTTTAGGTAACTTGAAC	For constructing *RSC1* specific Cas9-gRNA plasmid

Rvs167gRF	ACGAAACGAGTAAGCTCGTCTCAGGAAAGGATTGGTCAGAGTTTTAGAGCTAGAAATAGCAAGTT	For constructing *RVS167* specific Cas9-gRNA plasmid
Rvs167gRR	GACGAGCTTACTCGTTTCGTCCTCACGGACTCATCAGTCAGGATTTGATTTGTTTAGGTAACTTGAAC	For constructing *RVS167* specific Cas9-gRNA plasmid
Pmt6gRF	ACGAAACGAGTAAGCTCGTCCCAAGGCAGTGATCACCAGAGTTTTAGAGCTAGAAATAGCAAGTT	For constructing *PMT6* specific Cas9-gRNA plasmid
Pmt6gRR	GACGAGCTTACTCGTTTCGTCCTCACGGACTCATCAGCCAAGGTTTGATTTGTTTAGGTAACTTGAAC	For constructing *PMT6* specific Cas9-gRNA plasmid
rscdnF	CTCCAGAAGTTAATTCCTTCTCTGGTC	Amplify donor fragment of Rsc1^{G22V} from R1-3 genomic DNA
rscdnR	CTCAACGTGTTCTGGGTGTTCAC	Amplify donor fragment of Rsc1^{G22V} from R1-3 genomic DNA
rvsdnF	GAAGGTTGACCATAAGCAGGAGC	Amplify donor fragment of Rvs167^{G9R} from R3-24 genomic DNA
rvsdnR	GTAATCTCCAACGAAGATTCCAATG	Amplify donor fragment of Rvs167^{G9R} from R3-24 genomic DNA
pmtdnF	CGTCTCTAACCAGACTGATTACTTCG	Amplify donor fragment of Pmt6^{V57M} from R5-2 genomic DNA
pmtdnR	CATCGGAATGAATTTGCTCTGC	Amplify donor fragment of Pmt6^{V57M} from R5-2 genomic DNA
gRNA-F	GTGTCCTAGTTGTTATGTTTCGAGATG	Verification primer of N20 gRNA sequence
gRNA-R	CTTGCAACGTGACACCCTGTG	Verification primer of N20 gRNA sequence

Figure S1

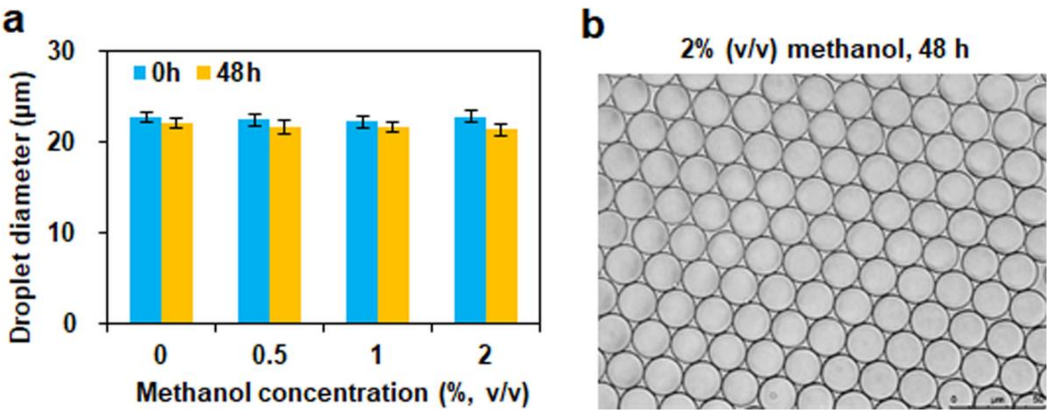

Fig. S1 Effects of methanol concentration on droplet stability.

a Mean diameter change of droplets containing different amounts of methanol. Droplets were off-chip incubated for 48 h at 30 °C. Data are presented as means ± standard deviations of three independent experiments. **b** Image of droplets containing 2% methanol at 48 h. Scale bar: 50 μm.

Figure S2

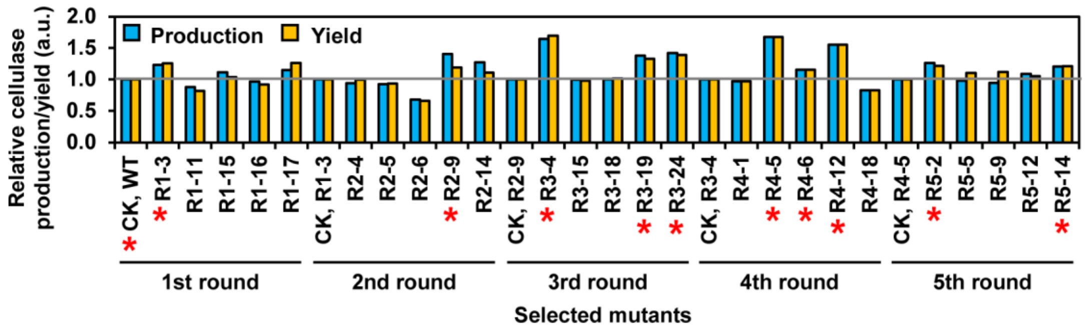

Fig. S2 Secondary validation of selected mutants by flask fermentation.

Top five cellulase hyperproducers from each round of five iterative ARTP mutagenesis and screening were evaluated for cellulase production by flask fermentation. Production is measured in units of cellulase activity per volume and the yield is cellulase activity per 1 OD_{600} unit of cells. Data were normalized to the starting strain of each round. The verified strains were indicated by red stars, and used for the final validation and genome resequencing analysis.

Figure S3

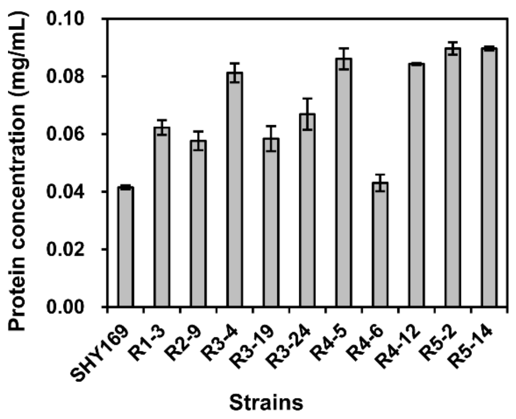

Fig. S3 Extracellular protein concentrations of the selected mutants.

Figure S4

```
CO    1 ATGGCTATTCCAAGATTCCCATCTATCTTCACTGCTGTTTTGTTCGCTGCTTCCTCCGCTTTGGCTGCTCCAGTCAACACTACTACCGAG
CO   91 GACGAAACTGCTCAAATTCCAGCTGAGGCTGTCATCGGTTACTCTGACCTGGAGGGTGACTTCGACGTTGCTGTCTTGCCATTCTCCAAC

CO  181 TCCACCAACAACGGTTTGTTGGAGGAGGCTGAAGCTGAAGCTGAACCTAAATTCATCAACACTACTATCGCTTCTATCGCTGCTAAGGAG
WT                                                                      ATGCGTTCCTCCCCCGTCCTCCGCACGGCC
                                                                        *    *  * *  *  **      * **
                Synthetic alpha-factor preproprotein leader sequence
                      (GenBank accession no. AY145833.1)

CO  271 GAGGGTGTTTCCCTCGAGAAAAGAGAGGCTGAAGCTTACGTAGAATTCGCCGACGGTAAATCCACTAGATACTGGGACTGCTGTAAACCA
WT      CTGGCCGCTGCCCTCC------------CCCTGGCCGCCCTCG---CTGCCGATGGCAAGTCGACCCGCTACTGGGACTGTTGCAAGCCG
          **  * * *****             *    **   * * *     ***** ** ** ** **  * *********** ** ** **

CO  361 TCCTGCTCTTGGCCAGGAAAGGCATCTGTCAATCAACCTGTCTTTGCCTGTTCCGCTAACTTCCAAAGAATCTCTGACCCAAACGTTAAG
WT      TCGTGCTCGTGGCCCGGCAAGGCCTCGGTGAACCAGCCCGTCTTCGCCTGCAGCGCCAACTTCCAGCGCATCAGCGACCCCAACGTCAAG
        ** ***** ***** ** ***** ** ** ** ** ** ***** *****   *** ********  * ***   ***** ***** ***

CO  451 TCCGGTTGTGATGGTGGTTCTGCTTACGCCTGCGCTGACCAAACCCCATGGGCTGTCAACGACAATTTCTCTTACGGTTTCGCTGCCACT
WT      TCGGGCTGCGACGGCGGCTCCGCCTACGCCTGCGCCGACCAGACCCCGTGGGCCGTCAACGACAACTTCTCGTACGGCTTCGCCGCCACG
        ** ** ** ** ** ** ** ** *********** ***** ***** ***** *********** ***** ***** ***** *****

CO  541 TCCATTTCCGGTGGTAACGAGGCCTCCTGGTGTTGCGGTTGTTACGAATTGACTTTCACCTCTGGTCCAGTTGCTGGAAAGACCATGGTT
WT      TCCATCTCGGGCGGCAACGAGGCCTCGTGGTGCTGTGGCTGCTACGAGCTGACCTTCACCTCGGGCCCCGTCGCTGGCAAGACCATGGTT
        ***** ** ** ** *********** ***** ** ** ** *****  **** ******** ** ** ** ***** ************

CO  631 GTCCAATCCACTTCTACTGGTGGTGACTTGGGTACTAACCACTTCGACTTGGCTATGCCAGGTGGTGGTGTTGGTATCTTCGACGGTTGT
WT      GTCCAGTCCACCTCGACCGGCGGCGACCTCGGCACCAACCACTTCGACCTGGCCATGCCCGGTGGTGGTGTCGGCATCTTCGACGGCTGC
        ***** ***** ** ** ** ** *** * ** ** ************ **** ***** *********** ** *********** **

CO  721 TCCCCACAATTCGGTGGTCTGGCTGGTGACAGATACGGTGGTGTTTCCTCTCGTTCTCAGTGTGACTCCTTCCCAGCAGCTCTTAAGCCT
WT      TCGCCCCAGTTCGGCGGCCTCGCCGGCGACCGCTACGGCGGCGTCTCGTCGCGCAGCCAGTGCGACTCGTTCCCCGCCGCCCTCAAGCCC
        ** ** ** ***** ** ** ** ** *** * ***** ** ** ** ** **    ***** ***** ***** ** ** ** *****

CO  811 GGTTGTTACTGGAGATTCGACTGGTTTAAAAACGCCGATAATCCAACTTTCACTTTCAGACAAGTTCAGTGTCCTTCCGAGTTGGTCGCT
WT      GGCTGCTACTGGCGCTTCGACTGGTTCAAGAACGCCGACAACCCGACCTTCACCTTCCGCCAGGTCCAGTGCCCGTCGGAGCTCGTCGCC
        ** ** ****** * *********** ** ******** ** ** ** ***** *** * ** ** ***** ** ** *** * *****

CO  901 AGAACCGGTTGCAGACGTAACGACGATGGTAACTTCCCAGTTTTCACTCCACCATCCGGTGGTCAATCCTCTTCTTCCTCCTCTTCCTCT
WT      CGCACCGGCTGCCGCCGCAACGACGACGGCAACTTCCCCGTCTTCACCCCTCCCTCGGGCGGTCAGTCCTCCTCGTCTTCCTCCTCCAGC
         * ***** *** * ** ******** ** ******** ** ***** ** ** ** ** ***** ***** ** ** ***** ***

CO  991 TCCGCTAAGCCAACTTCCACCTCTACTTCCACTACCTCCACCAAAGCCACTTCTACTACCTCCACCGCTTCTTCCCAAACCTCTTCCTCT
WT      AGCGCCAAGCCCACCTCCACCTCCACCTCGACCACCTCCACCAAGGCTACCTCCACCACCTCGACCGCCTCCAGCCAGACCTCGTCGTCC
          *** ***** ** ******** ** ** ** *********** ** ** ** ** ***** ***** **   *** ***** ** **

CO 1181 ACTGGTGGTGGTTGTGCCGCACAAAGATGGGCTCAGTGCGGTGGTATTGGTTTCTCCGGTTGTACTACCTGCGTCTCTGGTACTACCTGT
WT      ACCGGCGGCGGCTGCGCCGCCCAGCGCTGGGCGCAGTGCGGCGGCATCGGGTTCTCGGGCTGCACCACGTGCGTCAGCGGCACCACCTGC
        ** ** ** ** ** ***** **  * ***** ******** ** ** ** ***** ** ** ** ** ******   ** ** *****

CO 1271 AACAAGCAAAACGACTGGTACTCTCAGTGCTTGTAA
WT      AACAAGCAGAACGACTGGTACTCGCAGTGCCTTTAA
        ******** ************** ****** * ***
```

Fig. S4 Nucleotide sequence alignment between the wild-type (WT) and codon-optimized (CO) Staphylotrichum coccosporum stce1 genes.

The stce1 wild-type sequence was from GenBank accession no. AB248917.1.
